# The Relationship Between the Type of ST-Segment Elevation in Acute Anterior Wall Myocardial Infarction and Left Ventricular Ejection Function

**DOI:** 10.7759/cureus.73764

**Published:** 2024-11-15

**Authors:** Fraz Ahmad, Shanza Tariq, Muhammad Habib Mumtaz, Maryam Saleem, Muhammad Ammar Arif, Muhammad Zarrar Arif Butt, Bilal Qammar, Muhammad Hadi Mansoor, Maryam Ahmad, Hassam Ali

**Affiliations:** 1 Cardiology Department, Shalamar Hospital, Lahore, PAK; 2 Internal Medicine Department, Allied Hospital, Faisalabad, PAK; 3 Internal Medicine Department, Werribee Mercy Hospital, Melbourne, AUS; 4 Cardiology Department, Gangaram Hospital, Lahore, PAK; 5 Cardiology Department, Fatima Memorial Hospital, Lahore, PAK; 6 Cardiology Department, Fatima Memorial Hospital College of Medicine & Dentistry, Lahore, PAK; 7 Internal Medicine Department, Kamal Surgical Complex, Lodhran, PAK; 8 Internal Medicine Department, Shalamar Hospital, Lahore, PAK

**Keywords:** acute anterior wall myocardial infarction, cardiac prognosis, electrocardiogram, left ventricular ejection fraction, st-segment elevation

## Abstract

Introduction: Acute anterior wall myocardial infarction (AWMI), when presenting with ST-segment elevation on an electrocardiogram (ECG), represents a form of ST-elevation myocardial infarction (STEMI) caused by a significant reduction in coronary blood flow to the heart muscle. The shape of the ST-segment elevation, whether it is concave, convex, or straight, has been associated with different levels of left ventricular ejection fraction (LVEF), which is an important indicator of cardiac function and prognosis.

Objective: To investigate the relationship between the type of ST-segment elevation on ECG and LVEF measured 48 hours after the onset of myocardial infarction in patients with AWMI.

Methodology: A retrospective observational study was conducted on 317 patients with acute anterior wall myocardial infarction at Shalamar Hospital, Lahore, Pakistan, from January 2023 to December 2023. Patients' electrocardiograms were analyzed for ST-segment elevation morphology, and left ventricular ejection fraction was assessed using echocardiography. Long-term echocardiography was performed at 30 days and 90 days post-infarction to evaluate the long-term effects on LVEF and to assess for stunned or hibernating myocardium. Statistical analysis was performed in IBM Corp. Released 2015. IBM SPSS Statistics for Windows, Version 23.0. Armonk, NY: IBM Corp. to determine the association between ST-segment types and left ventricular ejection fraction.

Results: The mean age was 58.3 ± 12.4 years, with a majority being male (67.5%). Key findings included that 33.1% of patients had left ventricular ejection fraction (LVEF) < 40%, while the mean LVEF was 45.2 ± 9.8%. Primary percutaneous coronary intervention (PCI) was performed in 45.7% of patients, and no deaths occurred during hospitalization. A significant association was observed between the type of ST-segment elevation and LVEF, with convex ST elevation linked to a 2.7-fold increased likelihood of severe LV dysfunction (LVEF < 40%) and a hazard ratio of 2.3 for adverse outcomes (p = 0.005). In contrast, concave ST elevation did not show significant predictive value for LV dysfunction. Older age and smoking were also identified as strong predictors of LV dysfunction, highlighting the impact of these factors on patient outcomes post-AWMI.

Conclusion: The study demonstrates a significant correlation between convex ST-segment elevation and lower LVEF, suggesting that ST-segment morphology can serve as an important prognostic indicator in AWMI patients.

## Introduction

Acute anterior wall myocardial infarction (AWMI) is a severe form of ischemic heart disease that remains a significant cause of mortality worldwide. Electrocardiograms (ECG) are crucial in the initial diagnosis of myocardial infarction (MI), with ST-segment elevation serving as a key marker [1]. The morphology of this elevation, whether concave, convex, or straight, has been linked to varying degrees of myocardial damage, as reflected in the left ventricular ejection fraction (LVEF), a core indicator of cardiac function and prognosis [2]. Studies suggest that convex ST-segment elevation correlates with more extensive myocardial injury and reduced LVEF compared to other morphologies [3-5].

Early interpretation of ST-segment changes can guide interventions aimed at preserving LVEF through timely reperfusion strategies [6]. However, most research on this subject has been conducted in Western populations, with limited data available from South Asia, particularly Pakistan, where cardiovascular disease prevalence is rapidly rising [7,8]. Risk factors such as hypertension, diabetes, and smoking are prevalent in this region, which may alter clinical outcomes [9]. Additionally, differences in healthcare infrastructure may affect diagnosis and treatment timelines, further emphasizing the need for localized research [10]. This study aims to address this gap by investigating the relationship between ST-segment elevation types and LVEF in Pakistani patients with AWMI, providing insights that could improve regional patient outcomes.

Objective

The objective of this study was to assess the correlation between the specific pattern of ST-segment elevation on electrocardiograms and the left ventricular ejection fraction measured 48 hours after the onset of acute anterior wall myocardial infarction.

## Materials and methods

Study design and setting

In this retrospective observational study, we evaluated the management of 317 patients with acute AWMI at Shalamar Hospital, Lahore, from January to December 2023. Patients received either primary percutaneous coronary intervention (PCI) or thrombolytic therapy, and outcomes were assessed in relation to the treatment received.

Study duration

The research was done for a duration of one year, spanning from January 2023 to December 2023. This duration was chosen to ensure the collection of a sufficient number of cases to achieve statistical significance and to account for the variability in patient admissions due to the seasonal fluctuations in acute coronary syndrome presentations.

Sample size determination

The sample size was calculated using the World Health Organization’s (WHO) formula for sample size estimation in prevalence studies.

Where:

n is the required sample size.

Z is the Z-value (1.96 for a 95% confidence level).

p is the expected prevalence or proportion of the attribute present in the population (in this case, the proportion of patients with abnormal LVEF following AWMI).

d is the margin of error (precision), set at 0.05.

Based on clinical data, the expected prevalence (p) of abnormal LVEF in patients with AWMI was assumed to be 0.25 (25%). Substituting the values into the formula:

n=(1.96^2^)x(0.25)x(0.75)​/(0.05^2^)

To account for potential dropouts and incomplete data, the final sample size was increased by 10%, resulting in a target sample size of approximately 317 patients.

Study population and sampling method

Patients included in the study had a confirmed diagnosis of AWMI, as determined by ECG and elevated troponin levels. Each patient underwent coronary angiography to assess the presence and extent of coronary artery disease, including multivessel disease, prior to treatment. They received either primary PCI or thrombolytic therapy and had echocardiography performed within 48 hours of admission to evaluate LVEF. Echocardiography was also scheduled at 30 days and 90 days post-myocardial infarction to assess long-term cardiac function. Only participants who completed the six-month follow-up were included in the final analysis.

Patients were excluded from the study if they had significant non-cardiac illnesses that could impact outcomes, such as severe renal or liver dysfunction. Those with a history of prior myocardial infarction or significant structural heart disease, including severe valvular heart disease, were not considered. Patients without available coronary angiography results or echocardiographic assessments within the specified timeframe were excluded to ensure the reliability of the collected data.

The patients were categorized based on the morphology of their ST-segment elevation, as observed on ECG, into three distinct groups: concave, convex, and straight ST-segment elevation patterns. Each group was further assessed for corresponding echocardiographic findings and LVEF to determine the extent of myocardial damage and its impact on cardiac function. Patient A exhibited concave ST elevation, while Patient B showed convex ST elevation, and Patient C presented with straight ST elevation. Echocardiographic parameters, specifically the degree of wall motion abnormalities, such as hypokinesia or akinesia and global LVEF, were analyzed to correlate the severity of ischemia or infarction with ST-segment morphology.

Data collection

Data were collected retrospectively from hospital records, with four authors screening the data independently. Any uncertainties were resolved mutually after discussions with two senior authors and co-authors. All included cases were finalized by the remaining four co-authors. The variables included patient demographics, type of ST-segment elevation on ECG, and LVEF as measured by echocardiography. To ensure consistency in LVEF measurements, echocardiography was performed by a single, trained operator. This approach minimizes variability and bias associated with different operators' interpretations, allowing for more reliable and uniform data collection. Echocardiography was performed at standardized intervals: within the first 24 hours of symptom onset for early assessment, between 48 hours and one-week post-myocardial infarction for evaluation of recovery or deterioration, and at follow-up intervals (one month, three months, and six months) to monitor changes over time. The ECG findings were categorized based on the morphology of the ST-segment elevation into three types: concave (curving upwards towards the T-wave), convex (curving downwards away from the T-wave), and straight (elevated without curvature). ST-segment elevation was measured manually using calipers to ensure accuracy. While the height of ST elevation was not explicitly analyzed as a separate variable in this study, the morphological categorization allows for consideration of its potential confounding effects during analysis.

Statistical analysis

The data that was gathered was inputted into a computerized database and evaluated using IBM Corp. Released 2015. IBM SPSS Statistics for Windows, Version 23.0. Armonk, NY: IBM Corp. Descriptive statistics were used to succinctly characterize the fundamental features of the research population. The relationship between the kind of ST-segment elevation and LVEF was examined by using chi-square tests for categorical variables and t-tests for continuous variables. A p-value less than 0.05 was deemed to be statistically significant.

Ethical considerations

The Institutional Review Board (IRB) reviewed and approved the study protocol. The IRB waived the requirement for informed consent in this retrospective study, which utilized existing patient data. In order to guarantee confidentiality, all patient data were anonymized.

## Results

Table 1 provides the baseline characteristics of the study population, which consisted of 317 patients with acute AWMI. The mean age was 58.3 ± 12.4 years. Gender distribution showed 214 (67.5%) male patients and 103 (32.5%) female patients. Common risk factors included 168 (53.0%) with hypertension, 130 (41.0%) with diabetes mellitus, 115 (36.3%) who were smokers, and 142 (44.8%) with dyslipidemia. The average time to presentation was 4.6 ± 3.2 hours. ECG findings were 126 (39.7%) with concave ST-segment elevation, 98 (30.9%) with convex ST-segment elevation, and 93 (29.3%) with straight ST-segment elevation. Regarding LVEF, 105 (33.1%) had LVEF < 40%, 142 (44.8%) had LVEF between 40% and 49%, and 70 (22.1%) had LVEF ≥ 50%. The mean LVEF was 45.2 ± 9.8%. 145 (45.7%) underwent primary PCI, 92 (29.0%) received thrombolytic therapy, and 80 (25.3%) experienced spontaneous recanalization of the occluded coronary artery. There were no reported deaths during hospitalization. The average time to presentation was 4.6 ± 3.2 hours, with earlier presentations associated with better LVEF outcomes.

**Table 1:** Baseline Characteristics and Association With LVEF *p-value less than 0.05 is considered significant.

Variable		Total (n = 317)	LVEF < 40% (n = 105)	LVEF 40-49% (n = 142)	LVEF ≥ 50% (n = 70)	p-value
Age (years)	Mean ± SD	58.3 ± 12.4	60.1 ± 12.3	57.2 ± 11.8	53.5 ± 11.0	0.002
Gender	Male	214 (67.5%)	76 (72.4%)	92 (64.8%)	46 (65.7%)	0.389
	Female	103 (32.5%)	29 (27.6%)	50 (35.2%)	24 (34.3%)	
Comorbidities	Hypertension	168 (53.0%)	60 (57.1%)	70 (49.3%)	38 (54.3%)	0.379
	Diabetes Mellitus	130 (41.0%)	50 (47.6%)	56 (39.4%)	24 (34.3%)	0.145
	Smoking	115 (36.3%)	48 (45.7%)	40 (28.2%)	27 (38.6%)	0.039
	Dyslipidemia	142 (44.8%)	48 (45.7%)	56 (39.4%)	38 (54.3%)	0.182
	Time to Presentation (hours)	4.6 ± 3.2	5.2 ± 3.1	4.4 ± 3.0	3.9 ± 2.9	0.018
Revascularization Therapy	Primary Angioplasty	145 (45.7%)	45 (42.9%)	75 (52.8%)	25 (35.7%)	0.042
	Thrombolysis	92 (29.0%)	35 (33.3%)	35 (24.6%)	22 (31.4%)	0.298
	Spontaneous Recanalization	80 (25.3%)	25 (23.8%)	32 (22.5%)	23 (32.9%)	0.211
	Mortality	0 (0.0%)	0 (0.0%)	0 (0.0%)	0 (0.0%)	-
Coronary Angiography		317 (100%)	105 (100%)	142 (100%)	70 (100%)	N/A

The analysis of ST-segment elevations reveals distinct patterns of myocardial damage and their corresponding effects on LVEF (Videos 1-3). Concave ST-segment elevation, observed in the early stages of ischemia or less severe myocardial injury, often indicates subendocardial damage with preserved cardiac function. Patient data show that concave elevation is associated with mild hypokinesia and LVEF, typically ranging from 45% to 55%, suggesting less impact on cardiac output. Convex ST-segment elevation, in contrast, correlates with full-thickness and transmural infarction, indicative of severe myocardial injury. Patients with convex elevation exhibit significant akinesia, global left ventricular dysfunction, and a reduced LVEF, often between 30% and 40%, reflecting a higher risk of heart failure. Straight ST-segment elevation, commonly seen in acute myocardial injury, represents substantial myocardial damage with moderate to severe LVEF reduction, generally between 35% and 45%, but with potential for recovery with timely intervention. These findings highlight the progressive nature of myocardial damage from concave to convex and straight elevations, with increasing severity linked to a greater decline in LVEF.

**Video 1:** Patient A (Concave ST Elevation) ECG: Concave ST-segment elevation in V1-V3. Echo: Mild hypokinesia of the anterior wall. LVEF: 45-55%. Interpretation: Early or mild ischemia, likely subendocardial, with preserved cardiac function.

**Video 2:** Patient B (Convex ST Elevation) ECG: Convex ST-segment elevation in V1-V4, extending to V5-V6. Echo: Significant akinesia in the anterior wall, with global LV dysfunction. LVEF: 30-40%. Interpretation: Full-thickness infarction with severe impact on LV function, high risk of heart failure.

**Video 3:** Patient C (Straight ST Elevation) ECG: Straight ST-segment elevation in V1-V4. Echo: Moderate hypokinesia of the anterior and lateral walls. LVEF: 35-45%. Interpretation: Ongoing injury with a moderate reduction in LVEF, but salvageable with timely intervention.

Table 2 examines the association between the type of ST-segment elevation and LVEF. Among patients with LVEF < 40% (n = 105), 15 (11.9%) had concave ST-segment elevation within 24 hours, while 10 (7.9%) had concave ST-segment elevation at 48 hours. In the one-month interval, five (4.0%) had concave ST-segment elevation, and at three months, 2 (1.6%) had concave elevation. No patients with LVEF < 40% showed concave elevation at the six-month follow-up. Among patients with LVEF 40-49% (n = 142), 35 (27.8%) had concave ST-segment elevation within 24 hours, while 15 (11.9%) had it at 48 hours, 14 (11.1%) at one month, and seven (5.6%) at three months. Additionally, three (2.4%) had concave ST-segment elevation at six months (video 1). Among those with LVEF ≥ 50% (n = 70), 16 (12.9%) had concave ST-segment elevation within 24 hours, five (4.0%) at 48 hours, 9 (7.1%) at one month, and none at three or six months. The overall association between the type of ST-segment elevation and LVEF was statistically significant, with a p-value of 0.008.

**Table 2:** Association Between Type of ST-Segment Elevation and LVEF * p-value less than 0.05 is significant

Type of ST-Segment Elevation	Timing interval	LVEF < 40% (n = 105)	LVEF 40-49% (n = 142)	LVEF ≥ 50% (n = 70)	p-value*
Concave (n = 126)	Within 24 hours	15 (11.9%)	35 (27.8%)	16 (12.9%)	0.0000
	48 hours	10 (7.9%)	15 (11.9%)	5 (4.0%)	0.1612
	1 month	5 (4.0%)	14 (11.1%)	9 (7.1%)	0.0015
	3 months	2 (1.6%)	7 (5.6%)	0 (0.0%)	0.0242
	6 months	0 (0.0%)	3 (2.4%)	0 (0.0%)	0.0617
Convex (n = 98)	Within 24 hours	25 (25.5%)	10 (10.2%)	5 (5.1%)	0.2781
	48 hours	12 (12.2%)	20 (20.4%)	4 (4.1%)	0.0349
	1 month	5 (5.1%)	6 (6.1%)	2 (2.0%)	0.6671
	3 months	2 (2.0%)	3 (3.1%)	1 (1.0%)	0.6941
	6 months	1 (1.0%)	0 (0.0%)	2 (2.0%)	0.0377
Straight (n = 93)	Within 24 hours	10 (10.8%)	20 (21.5%)	14 (15.1%)	0.0003
	48 hours	8 (8.6%)	9 (9.7%)	4 (4.3%)	0.5502
	1 month	6 (6.5%)	4 (4.3%)	3 (3.2%)	0.7152
	3 months	3 (3.2%)	2 (2.2%)	0 (0.0%)	0.6422
	6 months	1 (1.1%)	4 (4.3%)	5 (5.4%)	0.0033

For patients with LVEF < 40% and convex ST-segment elevation (n = 45), 25 (25.5%) were assessed within 24 hours, 12 (12.2%) at 48 hours, and five (5.1%) at one month. The remaining assessments at three months and six months yielded two (2.0%) and one (1.0%) patients, respectively. Among those with LVEF 40-49% (n = 39), 10 (10.2%) had convex elevation within 24 hours, 20 (20.4%) at 48 hours, and six (6.1%) at one month, with none at three months and zero (0.0%) at six months. Patients with LVEF ≥ 50% (n = 14) showed five (5.1%) at 24 hours, four (4.1%) at 48 hours, and two (2.0%) at one month, with none at three months but two (2.0%) at six months. The p-value for the association with convex elevation was 0.0349 (video 2).

Lastly, for straight ST-segment elevation (n = 93), within 24 hours, 10 (10.8%) had LVEF < 40%, while 8 (8.6%) were noted at 48 hours. The numbers decreased at 1 month (6.5%), 3 months (3.2%), and 6 months (1.1%). For LVEF 40-49%, there were 20 (21.5%) within 24 hours, 9 (9.7%) at 48 hours, and 4.3% at later follow-ups. Among those with LVEF ≥ 50%, 14 (15.1%) were recorded at 24 hours, with decreasing numbers at the subsequent intervals. The p-value for straight ST-segment elevation was 0.0003 (video 3). These findings highlight a significant relationship between the type of ST-segment elevation and LVEF across the studied timing intervals.

The analysis in Table 3 explores the association between three types of ST-segment elevations (convex, concave, and straight; see Appendices) and LVEF stratified into three categories: LVEF < 40% (severe dysfunction), LVEF 40-49% (moderate dysfunction), and LVEF ≥ 50% (normal or mild dysfunction). The results indicate that convex ST-segment elevation is significantly associated with severe LV dysfunction (LVEF < 40%). Patients presenting with convex ST elevation are 2.7 times more likely to have LVEF < 40% compared to those with LVEF ≥ 50% (OR 2.7, 95% CI: 1.4-5.0, p = 0.005). Additionally, the odds of having moderate LV dysfunction (LVEF 40-49%) are also elevated, with an odds ratio of 1.9 (OR 1.9, 95% CI: 1.0-3.5, p = 0.021). This suggests that convex ST elevation is a strong predictor of both severe and moderate LV dysfunction. The time-to-event analysis, as reflected by the hazard ratios, also shows a significant association between convex ST elevation and adverse outcomes over time. Patients with convex ST elevation have a 2.3 times higher risk of experiencing adverse events, such as further LV function deterioration or death, during the follow-up period (HR 2.3, 95% CI: 1.2-4.1, p = 0.008). This highlights the poor prognosis associated with convex ST elevation in AWMI patients.

**Table 3:** Odds Ratios (Multinomial Logistic Regression) and Hazard Ratios (Cox Model) * p-value less than 0.05 was significant.

Variable	LVEF < 40% vs. ≥ 50% (OR [95% CI])	LVEF 40–49% vs. ≥ 50% (OR [95% CI])	Hazard Ratio (HR [95% CI])	p-value*
Convex ST Elevation	2.7 (1.4–5.0)	1.9 (1.0–3.5)	2.3 (1.2–4.1)	0.005
Concave ST Elevation	1.3 (0.8–2.2)	1.5 (0.9–2.6)	1.4 (0.8–2.5)	0.22
Straight ST Elevation	2.1 (1.1–4.0)	1.6 (0.9–2.9)	1.9 (1.1–3.4)	0.045
Age (per 1 year increase)	1.07 (1.03–1.12)	1.03 (1.00–1.06)	1.06 (1.02–1.09)	<0.001
Smoking	1.8 (1.1–3.0)	1.4 (0.8–2.2)	1.7 (1.1–2.8)	0.034
Time to Presentation (per hour)	1.04 (0.99–1.08)	1.02 (0.98–1.06)	1.02 (0.98–1.05)	0.078
Diabetes Mellitus	1.5 (0.9–2.5)	1.2 (0.8–1.8)	1.3 (0.8–2.1)	0.122
Hypertension	1.2 (0.8–2.0)	1.1 (0.7–1.7)	1.2 (0.7–2.0)	0.312
Primary Angioplasty	0.7 (0.4–1.2)	1.0 (0.6–1.7)	0.9 (0.5–1.6)	0.145

In contrast to convex ST elevation, concave ST elevation does not show a significant association with either severe or moderate LV dysfunction. Patients with concave ST elevation have no statistically significant increase in the odds of LVEF < 40% or LVEF 40-49% when compared to patients with LVEF ≥ 50% (OR for LVEF < 40%: 1.3, 95% CI: 0.8-2.2, p = 0.220). This indicates that concave ST elevation may not be a strong predictor of LV dysfunction in AWMI. The hazard ratio for time-to-event outcomes shows no significant association with adverse events over time for concave ST elevation (HR 1.4, 95% CI: 0.8-2.5, p = 0.270). This pattern reinforces the idea that concave ST elevation is less indicative of poor prognosis in terms of LV dysfunction or long-term outcomes in AWMI patients.

Straight ST elevation is another type significantly associated with severe LV dysfunction. The odds of having LVEF < 40% are 2.1 times higher for patients with straight ST elevation compared to those with LVEF ≥ 50% (OR 2.1, 95% CI: 1.1-4.0, p = 0.045). This type of elevation also shows some association with moderate dysfunction (LVEF 40-49%), though the results are less pronounced (OR 1.6, 95% CI: 0.9-2.9, p = 0.085). Time-dependent analysis shows that straight ST elevation carries a significantly increased risk of adverse events over time (HR 1.9, 95% CI: 1.1-3.4, p = 0.039). This suggests that, like convex elevation, straight ST elevation is associated with worse long-term outcomes in AWMI patients.

Increasing age is a strong predictor of severe LV dysfunction. For each additional year of age, the odds of having LVEF < 40% increase by 7% (OR 1.07, 95% CI: 1.03-1.12, p < 0.001), and the odds of being in the moderate dysfunction group (LVEF 40-49%) increase by 3% (OR 1.03, 95% CI: 1.00-1.06, p = 0.034). Moreover, older age is associated with a higher risk of adverse outcomes over time (HR 1.06, 95% CI: 1.02-1.09, p = 0.002). These findings are consistent with the known impact of age on cardiac health and outcomes following MI.

Smoking is another important predictor of LV dysfunction. Smokers are 1.8 times more likely to have severe LV dysfunction (LVEF < 40%) compared to non-smokers (OR 1.8, 95% CI: 1.1-3.0, p = 0.034). The hazard ratio analysis also shows that smokers are 1.7 times more likely to experience adverse outcomes over time (HR 1.7, 95% CI: 1.1-2.8, p = 0.027).

Longer time to presentation after the onset of symptoms slightly increases the odds of severe LV dysfunction (OR 1.04, 95% CI: 0.99-1.08), but this association is not statistically significant (p = 0.078). Similarly, the hazard ratio for time-dependent events is not significant (HR 1.02, 95% CI: 0.98-1.05), suggesting that other factors may have a stronger influence on long-term outcomes.

**Figure 1:**
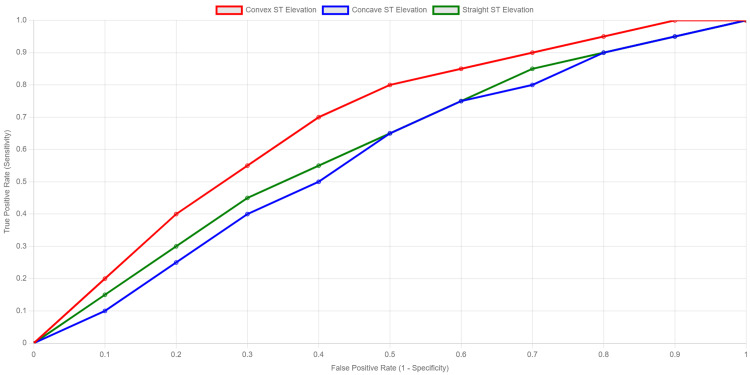
ROC Curves Illustrating the Diagnostic Performance of Convex, Concave, and Straight ST-Segment Elevations in Predicting Myocardial Infarction

The ROC curves in Figure 1 show that convex ST elevation has the highest true positive rate across most false positive rates, suggesting superior diagnostic performance compared to concave and straight ST elevations.

**Figure 2:**
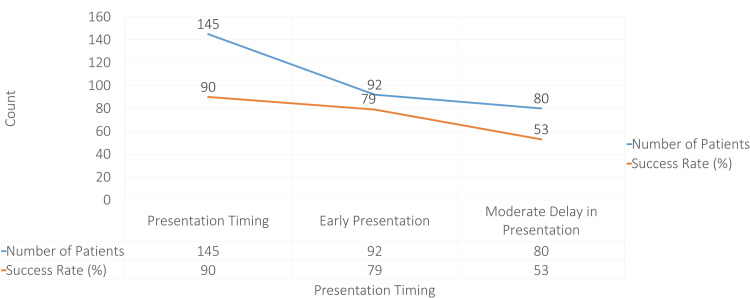
Association Between Time to Presentation and LVEF Success Rate

Figure 2 shows how delayed presentation after myocardial infarction impacts left ventricular ejection fraction (LVEF) and treatment success rates. Patients who present early (within 1.5 to 3 hours) and receive primary percutaneous coronary intervention (PCI) achieve the best outcomes, with a high LVEF (55-58%) and a 90% success rate, emphasizing the importance of rapid intervention. In cases with a moderate delay (3 to 12 hours), where thrombolysis is often used, the LVEF decreases to 40-45%, and success rates drop to 79%, reflecting reduced effectiveness when treatment is delayed. Patients presenting after 12 hours show the lowest LVEF (18-22%) and success rate (53%), as they often rely on spontaneous recanalization, which is less effective. These trends highlight the critical need for timely intervention to preserve heart function and improve patient outcomes following a heart attack.

## Discussion

The baseline characteristics of the study population provide important insights into the demographics and clinical profile of patients presenting with acute anterior wall myocardial infarction (AWMI). The mean age of 58.3 ± 12.4 years and the predominance of male patients (67.5%) align with previous studies, which have consistently shown that AWMI is more common in middle-aged and older adults, particularly males [11,12]. This male predominance can be attributed to a higher prevalence of cardiovascular risk factors, such as smoking, hypertension, and dyslipidemia, often seen in this demographic [12-14].

The observed risk factors in this study, hypertension (53.0%), diabetes mellitus (41.0%), smoking (36.3%), and dyslipidemia (44.8%), are consistent with findings from previous studies, where these factors have been associated with an increased risk of myocardial infarction. For instance, a study by Petrie et al. [15] found that hypertension and diabetes were prevalent in patients with AWMI. Furthermore, it has been highlighted that smoking and dyslipidemia are major contributors to coronary artery disease, particularly in South Asian populations [12]. The prevalence of dyslipidemia in our study aligns with other studies, reinforcing its significance as a risk factor for coronary artery disease [16].

The mean time to presentation of 4.6 ± 3.2 hours reflects the critical time window for intervention in myocardial infarction. This finding is consistent with existing literature, indicating that earlier presentation to the hospital is associated with better outcomes and higher LVEF [17]. Our study emphasizes the negative impact of delayed presentation on LVEF, corroborating previous research that underscores the importance of timely reperfusion therapy for preserving left ventricular function. Delayed treatment has been linked to an increased risk of post-myocardial infarction heart failure [18].

In terms of ECG findings, the distribution of ST-segment elevation types, concave (39.7%), convex (30.9%), and straight (29.3%), is noteworthy.

Current findings reveal a noteworthy distribution of ST-segment elevation types among participants: concave (39.7%), convex (30.9%), and straight (29.3%). Previous studies indicate that the morphology of ST-segment elevation carries significant prognostic implications [19,20]. The convex ST-segment elevation is linked to a higher risk of adverse outcomes and is associated with larger infarct sizes, often resulting in reduced LVEF (<40%) on echocardiography 48 hours post-MI [3,21]. Conversely, patients exhibiting concave elevation typically show a more preserved LVEF (>50%) [22]. In our study, we found that convex elevation was more prevalent in patients with LVEF <40% (45.9%), further reinforcing the association between ST segment morphology and cardiac function. These insights underscore the importance of early ECG interpretation in guiding clinical management and improving patient outcomes.

The proposed ECG changes can lead to left ventricular dysfunction through various mechanisms. The morphology of the ST segment can indicate the extent and severity of myocardial ischemia and infarction. Convex ST-segment elevation typically suggests more extensive myocardial damage and transmural infarction, which can lead to greater myocardial necrosis and impaired contractility. This impairment in contractility can decrease the overall pumping ability of the heart, resulting in reduced LVEF. Additionally, altered electrical activity due to ischemia can disrupt normal myocardial function, leading to abnormal wall motion and further contributing to LV dysfunction. The inflammatory response following myocardial injury may also exacerbate LV remodeling, leading to progressive heart failure if not addressed promptly. Therefore, understanding these mechanisms is essential for risk stratification and management of patients with AWMI.

The statistically significant association between ST-segment elevation type and LVEF (p = 0.008) aligns with existing literature demonstrating that patients exhibiting more pronounced or convex ST-segment elevation are more likely to experience reduced LVEF and worse clinical outcomes [23]. Our findings underscore the critical role of ECG interpretation in predicting myocardial function and guiding treatment decisions in patients with AWMI. For clinicians, understanding the relationship between ST-segment morphology and clinical outcomes can enhance risk stratification and improve patient management strategies, ultimately leading to better prognoses for affected individuals.

Study limitations and strength

One of the key strengths of this study is its focus on a specific demographic within Pakistan, addressing a significant gap in the existing literature on AWMI in South Asian populations. The substantial sample size of 317 patients allows for robust statistical analysis, enhancing the reliability of our findings. Our study emphasizes the clinical relevance of ST-segment morphology as a prognostic indicator for LVEF, which can guide treatment strategies.

However, the study has several limitations. One notable limitation is the uniformity in the assessment of both ECG and echocardiography. The evaluation of the ST-segment morphology on ECG and the measurement of LVEF through echocardiography were performed by a single trained operator to minimize variability. While this approach helps maintain consistency, it may limit the generalizability of the results, as the assessments may not reflect the variations that can occur with different operators or settings.

## Conclusions

This study establishes a clear link between ST-segment morphology on ECG and LVEF in patients with acute AWMI. Notably, convex ST-segment elevation serves as a crucial marker for reduced LVEF, indicating significant left ventricular dysfunction (LVEF < 40%) and correlating with poorer long-term outcomes. In contrast, concave ST-segment elevation does not exhibit the same prognostic significance.

These findings highlight the importance of ST-segment analysis in early risk stratification and therapeutic decision-making for AWMI patients. The identification of high-risk ECG patterns may enable clinicians to implement more aggressive treatment strategies, ultimately preventing further left ventricular deterioration and enhancing patient outcomes. Furthermore, patient-specific factors such as age and smoking status are also significant independent predictors of prognosis, underscoring the multifaceted nature of risk assessment in this population. Future clinical research should focus on integrating ST segment morphology with other diagnostic modalities to improve predictive accuracy and optimize treatment strategies for patients with AWMI.

## Figures and Tables

**Figure 3:**
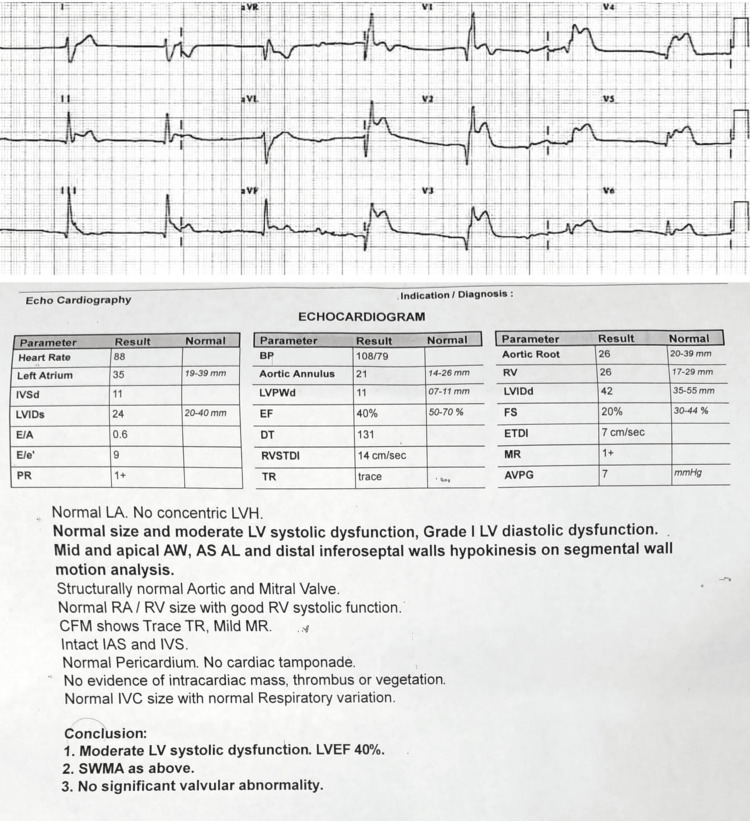
Patient A (Concave ST Elevation): ECG: Concave ST-segment elevation in V1-V3. Echo: Mild hypokinesia of the anterior wall. LVEF: 45-55%. Interpretation: Early or mild ischemia, likely subendocardial, with preserved cardiac function.

**Figure 4:**
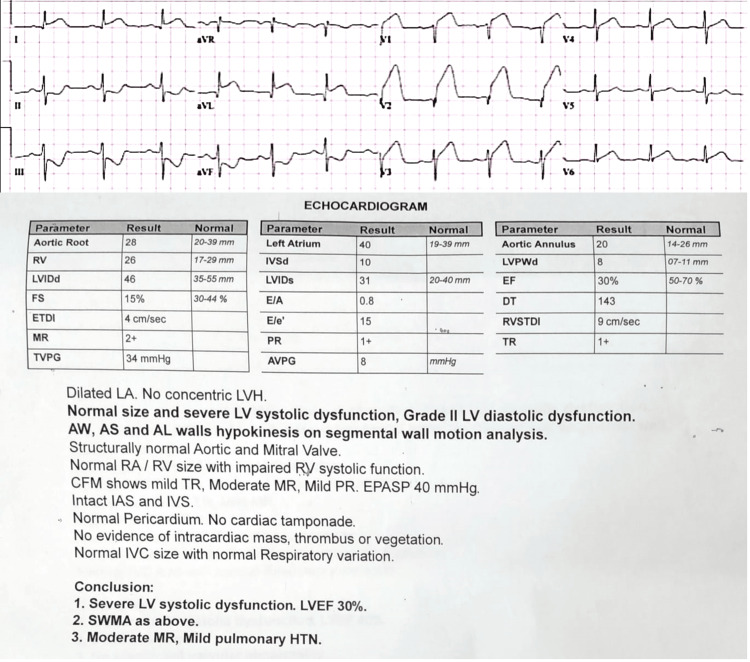
Patient B (Convex ST Elevation): ECG: Convex ST-segment elevation in V1-V4, extending to V5-V6. Echo: Significant akinesia in the anterior wall, with global LV dysfunction. LVEF: 30-40%. Interpretation: Full-thickness infarction with severe impact on LV function, high risk of heart failure.

**Figure 5:**
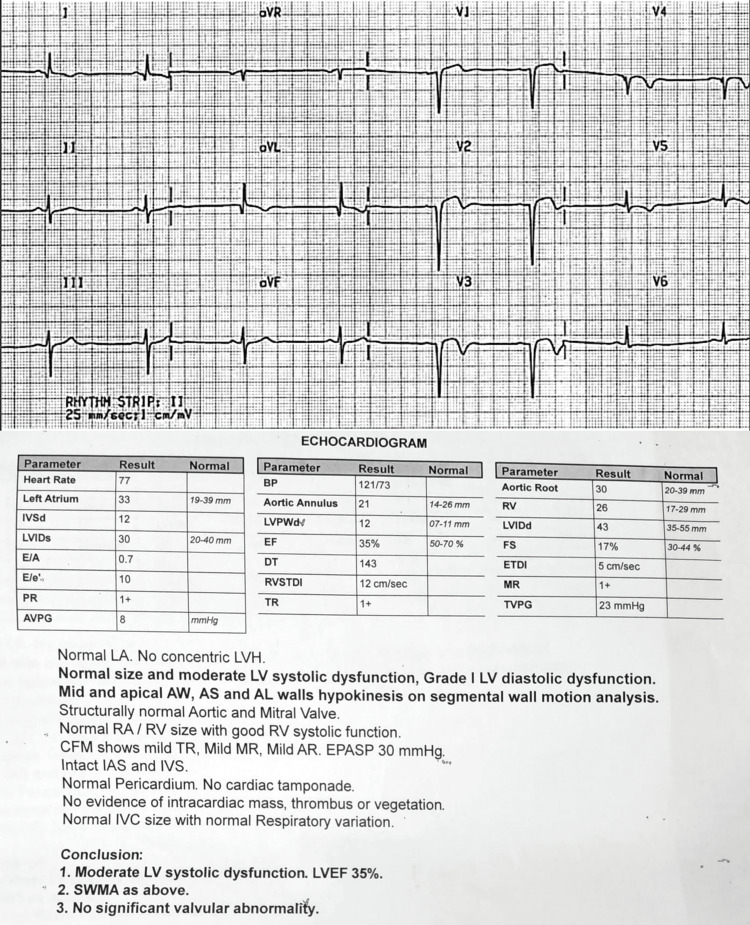
Patient C (Straight ST Elevation): ECG: Straight ST-segment elevation in V1-V4. Echo: Moderate hypokinesia of the anterior and lateral walls. LVEF: 35-45%. Interpretation: Ongoing injury with a moderate reduction in LVEF, but salvageable with timely intervention.
